# Potential Impact of Diabetes and Obesity on Alveolar Type 2 (AT2)-Lipofibroblast (LIF) Interactions After COVID-19 Infection

**DOI:** 10.3389/fcell.2021.676150

**Published:** 2021-07-08

**Authors:** Marjan Nouri-Keshtkar, Sara Taghizadeh, Aisan Farhadi, Aysan Ezaddoustdar, Samira Vesali, Roya Hosseini, Mehdi Totonchi, Azam Kouhkan, Chengshui Chen, Jin-San Zhang, Saverio Bellusci, Yaser Tahamtani

**Affiliations:** ^1^Faculty of Sciences and Advanced Technologies in Biology, University of Science and Culture, Tehran, Iran; ^2^Department of Stem Cells and Developmental Biology, Cell Science Research Center, Royan Institute for Stem Cell Biology and Technology, ACECR, Tehran, Iran; ^3^Key Laboratory of Interventional Pulmonology of Zhejiang Province, Department of Pulmonary and Critical Care Medicine, The First Affiliated Hospital of Wenzhou Medical University, Wenzhou, China; ^4^Excellence Cluster Cardio-Pulmonary System, Justus Liebig University Giessen, Giessen, Germany; ^5^Faculty of Veterinary Medicine, University of Tehran, Tehran, Iran; ^6^Reproductive Epidemiology Research Center, Royan Institute for Reproductive Biomedicine, ACECR, Tehran, Iran; ^7^Department of Genetics, Reproductive Biomedicine Research Center, Royan Institute for Reproductive Biomedicine, ACECR, Tehran, Iran

**Keywords:** SARS-CoV-2, diabetes mellitus, obesity, inflammation, Lipofibroblasts, angiotensin-converting enzyme 2, alveolar epithelial type 2 cells, myofibroblast

## Abstract

Severe acute respiratory syndrome coronavirus 2 (SARS-CoV-2), a new emerging respiratory virus, caused evolving pneumonia outbreak around the world. In SARS-Cov-2 infected patients, diabetes mellitus (DM) and obesity are two metabolic diseases associated with higher severity of SARS-CoV-2 related complications, characterized by acute lung injury requiring assisted ventilation as well as fibrosis development in surviving patients. Different factors are potentially responsible for this exacerbated response to SARS-CoV-2 infection. In patients with DM, base-line increase in inflammation and oxidative stress represent preexisting risk factors for virus-induced damages. Such factors are also likely to be found in obese patients. In addition, it has been proposed that massive injury to the alveolar epithelial type 2 (AT2) cells, which express the SARS-CoV-2 receptor angiotensin-converting enzyme 2 (ACE2), leads to the activation of their stromal niches represented by the Lipofibroblasts (LIF). LIF are instrumental in maintaining the self-renewal of AT2 stem cells. LIF have been proposed to transdifferentiate into Myofibroblast (MYF) following injury to AT2 cells, thereby contributing to fibrosis. We hypothesized that LIF’s activity could be impacted by DM or obesity in an age- and gender-dependent manner, rendering them more prone to transition toward the profibrotic MYF status in the context of severe COVID-19 pneumonia. Understanding the cumulative effects of DM and/or obesity in the context of SARS-CoV-2 infection at the cellular level will be crucial for efficient therapeutic solutions.

## Introduction

Severe acute respiratory syndrome coronavirus 2 (SARS-CoV-2) is responsible for the ongoing global outbreak of the so-called “coronavirus disease 2019 (COVID-19)”-related pneumonia (Chen N. et al., 2020). In general, respiratory viruses boost lung injury by activating the cytokine storm, inflammation, massive epithelial/endothelial cell death, vascular leakage, and other pathological conditions (Khomich et al., 2018). Overt immune reactions trigger the surge in production of numerous cytokines causing respiratory diseases like pneumonitis and acute respiratory distress syndrome (ARDS). Overtime, repeated events of exacerbated immune responses, result in organ failure and death (Wang and Ma, 2008; Matthay et al., 2012). Following respiratory infections, activated alveolar macrophages release proinflammatory cytokines, leading to increased expression of cell adhesion molecules (CAMs) and vascular endothelial growth factor (VEGF) accompanied by increased permeability of the lung endothelium (Hiraiwa and van Eeden, 2014). Lung specimens from infected patients showed desquamation of pneumocytes, pulmonary edema and proteinaceous exudates, hyaline membrane formation, and alveolar wall thickening due to fibroblast proliferation accompanied by type II pneumocyte hyperplasia (Leng et al., 2020).

Among the pre-existing risk factors know to exacerbate COVID-19-induced pathogenic mechanisms in humans, diabetes mellitus (DM), and obesity (in relation with aging and gender) have been highlighted as predisposing factors for complications associated with sever COVID-19 pneumonia (summarized in Table 1; Cai et al., 2020; Muniyappa and Gubbi, 2020; Zhang et al., 2020). DM and obesity are clear manifestations of metabolic dysfunctions associated with cardiovascular diseases and hypertension (Gao et al., 2020; Stefan et al., 2020). Moreover, induction of inflammation in obese people through imbalanced production of adipokines, especially adiponectin (anti-inflammatory adipokine) and leptin (pro-inflammatory), by the adipose tissue increases the obesity-associated risk of severe COVID-19 complications (Assad and Sood, 2012; Messina et al., 2020; Salvator et al., 2020). Diabetic patients with uncontrolled glucose homeostasis have been reported to display poor immunity against viral infection (Hodgson et al., 2015). Indeed, the severity of COVID-19 infection in DM and obese patients correlates with unbalanced immune response, increased sensitivity to hyperinflammation and cytokine storm, as well as low level of viral clearance (Muniyappa and Gubbi, 2020).

**TABLE 1:** Relation between DM and obesity and risk of COVID-19-related failures.

Main purpose	Study type	Date^†^	Population/Location	Parameters	Key findings	References
Potential association between obesity and severe outcomes of COVID-19	Retrospective cohort	April 2020	Patients hospitalized with COVID-19/United States	Obesity, other chronic disease	Severe obesity (BMI^1^ ≥ 35 kg/m^2^) associated with ICU^2^ admission and IVM^3^	Kalligeros et al., 2020
Obesity significantly associated with respiratory failure and death	Retrospective cohort	February to April 2020	COVID-19 patients admitted to hospital/Italy	Obesity (BMI ≥ 30 kg/m^2^), demographic data, comorbidities, clinical and radiological examinations	Older obese patients more likely at risk for respiratory failure and death of COVID-19	Rottoli et al., 2020
Relation between obesity and SARS-CoV-2	Retrospective cohort	February to April 2020	patients admitted to intensive care for SARS-CoV-2/French	Moderate obesity (30 to <35 kg/m^2^)/severe obesity (≥35 kg/m^2^), epidemiological data, past medical history, treatments and clinical data	The proportion of patients who required IMV increased with male sex and BMI categories, greatest in patients with BMI > 35 kg/m^2^	Simonnet et al., 2020
Obesity as a risk factors of Severe illness in Patients with COVID-19	Retrospective	January, and February 2020	patients with COVID-19/Jiangsu, China	Obesity (BMI ≥ 28 kg/m^2^), clinical characteristics, complications, and outcomes of patients	Obesity as an independent risk factor of respiratory failure and acute respiratory distress syndrome	Wang J. et al., 2020
Higher BMI as a risk factor for progression to severe COVID-19	Cohort	January and February 2020	Hospitalized patients with COVID-19/Shenzhen, China	Obesity (BMI ≥ 28 kg/m^2^), epidemiological, clinical and laboratory characteristics	Obese patients had increased odds of progressing to severe COVID-19.	Cai et al., 2020
Relationship between overweight/obesity and COVID-19	Meta-Analysis	January to June 2020	COVID-19 patients/global	BMI, epidemiological and clinical characteristics, treatment outcomes	Patients with obesity were more at risk for COVID-19 positive, hospitalization, ICU admission, mortality	Popkin et al., 2020
Effect of obesity on outcomes in the COVID-19 hospitalizations	Meta-analysis	December 2019 to August 2020	COVID-19 hospitalized patients/global	BMI, treatment outcomes	COVID-19 patients with pre-existing obesity had higher risk of having worse outcomes.	Malik et al., 2020
Clinical characteristics and outcomes of Type 2 diabetes patients infected with COVID-19	Retrospective	January to March 2020	COVID-19 patients hospitalized with(out) diabetes ≥45 years/Hubei, China	Clinical, radiographic and laboratory features, complications, treatments, and clinical outcomes	Frequency/degree of abnormalities in CT^4^ chest scans markedly increased in COVID-19 patients with diabetes	Chen Y. et al., 2020
Risk factors for in-hospital mortality of COVID-19 patients with diabetes	Retrospective	January to March 2020	COVID-19 patients with(out) diabetes/Wuhan, China	Demographic data, underlying comorbidities, laboratory parameters on admission, CT scans	Diabetic patients with age ≥70 years and with hypertension had a higher hazard ratio for in-hospital death	Shi et al., 2020
Diabetes as a risk factor for the progression and prognosis of COVID-19	Retrospective	February 2020	SARS-Cov-2 patients hospitalized with(out) diabetes/Wuhan, China	Demographic data, medical history, laboratory findings, CT scans	Diabetes as a risk factor for a rapid progression in organ damage/deterioration and inflammatory storm	Guo et al., 2020
Independent effects of diabetes status, by type, on in-hospital death	Retrospective	March to May 2020	COVID-19 patients with(out) diabetes/England	Demographic and clinical data	Type 1 and type 2 diabetes associated with increased odds of in-hospital death with COVID-19	Barron et al., 2020
Glycemic characteristics and clinical outcomes of COVID-19 patients hospitalized	Retrospective cohort	March to April 2020	Patients hospitalized with laboratory-confirmed COVID-19/United States	Demographic, clinical and laboratory characteristics	Patients with uncontrolled hyperglycemia had higher median length of stay at hospital and particularly higher mortality rate.	Bode et al., 2020
Impact of DM on COVID-19 patients	Meta-analysis	January to April 2020	Confirmed COVID-19 patients/global	Treatment outcomes	Higher mortality and ICU admission risk in COVID-19 patients with diabetes compared to non-diabetics	Hussain et al., 2020
Association of diabetes with the clinical severity and in-hospital mortality from COVID-19	Meta-analysis	January to May 2020	COVID-19 patients admitted to hospital/global	Treatment outcomes	Pre-existing diabetes as a two to three times greater risk of severe/critical illness and in-hospital mortality	Mantovani et al., 2020

*^†^Date: Data collection time.*

*^1^Body mass index; ^2^Intensive care unit; ^3^Invasive mechanical ventilation; ^4^Computed tomography.*

Recruitment and normal functions of neutrophil and macrophage can be impaired in patients with DM (Hodgson et al., 2015). It is well established that innate and adaptive immune responses to viral infections and the subsequent bacterial infections in the lungs are disrupted by poor glycemic control in multiple ways (Carey et al., 2018; Ferlita et al., 2019). DM/obesity exerts a detrimental impact on the host immune system, increasing susceptibility to infections and extending their clinical manifestations (Talbot et al., 2012; Frydrych et al., 2018). These defects, such as T cell dysfunction, defective natural killer (NK) cells, and abnormal complement activity impair viral clearance (Nyambuya et al., 2020). Indeed, metabolic changes caused by persistent hyperglycemia in diabetic individuals (Brownlee, 2001; Tiwari et al., 2011) result in generation of higher amounts of superoxide and activate inflammatory pathways, which lead to immune system dysfunction (Hameed et al., 2015). A study on patients with type 2 diabetes demonstrated that neutrophils and monocytes release excessive proinflammatory cytokines such as TNF-α, IL-1β, and IL-8, which provide suitable conditions for pathogen invasions (Hatanaka et al., 2006). Besides alteration of the phenotypes and activity of NK cells, there is also downregulation of receptors on NK cells which recognize viruses (Berrou et al., 2013). In addition, it has been reported that serum level of inflammatory cytokines, including IL-6, in COVID-19 patients with diabetes is higher than those patients without diabetes resulting in increased susceptibility to COVID-19-related infections in diabetic patients (Guo et al., 2020). Interestingly, exposure of pulmonary epithelial cells to high level of glucose concentrations significantly elevated influenza virus replication and infection, suggesting that elevated levels of glucose can facilitate respiratory virus replication. To further complicate further the clinical situation, diabetic patients have a delay in clearance of SARS-CoV-2 which may lead to further injuries (Wang J. et al., 2020).

In addition, DM/obesity can increase the expression of angiotensin-converting enzyme 2 (ACE2, the main cell receptor for SARS-CoV-2) (Rao et al., 2020). It has been found that high fat diet-induced obesity in mouse increases ACE2 expression in lung epithelial cells, which can enhance the infection of AT2 cells by COVID-19 (Al Heialy et al., 2020). AT2 cells represent the main functional cells in the alveolar region by virtue of producing surfactant, critical for lowering/reducing surface tension to prevent alveolar collapse (Shao et al., 2020) as well as by serving as stem cells (Barkauskas et al., 2013). Therefore, massive AT2 injury leads to impaired lung function, and eventually cell death, especially in the context of pre-existing metabolic dysregulation (obesity/diabetes), gender (males versus females), and age (old versus young). However, the molecular mechanism(s) and the cell type(s) involved in the exacerbated response to SARS-CoV-2 in patients with pre-existing conditions are still largely unknown.

A better vision of the cumulative effects of DM/obesity and COVID-19 in the induction of the pathogenic mechanisms at the cellular level in the lung, such as in depth characterization of the destructive effects on the resident mesenchymal niche cells associated with AT2 cells, is essential for understanding the role of DM/obesity in increasing the risk of COVID-19-related complications and for further treatments and prevention strategies, and the detailed investigation of the destructive effects on the resident mesenchymal niche cells which are associated with AT2 cells.

Therefore, in the next paragraphs of this review, we first focus on the signaling pathways/molecules that have a role in the sensitivity of diabetic/obese patients to COVID-19-related infections. We then review the latest knowledge about the pathogenic mechanisms-related to COVID-19 at the cellular level. Finally, we discuss the AT2-LIF interactions in this condition and introduce new cell- and organoid based models, which can be utilized, to better characterized the status of such interactions at the cellular and molecular level (Bode et al., 2020).

## Destructive Pathways Triggered by SARS-CoV-2 in the Context of DM/Obesity

### General Pathways Triggered by SARS-CoV-2/DM

Diabetes mellitus/obesity can impact lung function as well as the integrity of the capillary endothelium (Goldman, 2003; Zheng et al., 2017; Roberts et al., 2018). Increased thickness of the capillary and epithelial basal lamina of the alveoli has been reported in the patients with DM (Pitocco et al., 2012). These damages are illustrated by the reduction in the pulmonary diffusing capacity for carbon monoxide, a reduction in the total lung capacity, and a lower forced vital capacity (Pitocco et al., 2012; Kolahian et al., 2019). Poorly controlled blood glucose levels and abnormalities in insulin function can cause damage in the lung through different mechanisms including inflammation and oxidative stress (Yang et al., 2011; Wu et al., 2017). On the other hand, infection of lung cells with respiratory viruses can lead to the activation of apoptotic or pyroptosis pathways, inflammation as well as enhanced reactive oxygen species (ROS) production (Khomich et al., 2018).

### Induced Oxidative Stress in Infected Diabetic Lung Cells

Hyperglycemia can induce oxidative stress, resulting in damage to the lipids, proteins and DNA. Experiments showed that in diabetic lung, content of malondialdehyde (MDA) was significantly increased, but the activity of superoxide dismutase (SOD), one of the key antioxidant enzymes, was decreased (Forgiarini Junior et al., 2010; Zhang et al., 2015). Hyperglycemia can induce different abnormalities in the lung, and vascular endothelial cell that can provoke virus-related damage (Goldman, 2003). These abnormalities include mitochondrial dysfunction, advanced glycation end products (AGEs) accumulation as well as the activation of the polyol pathway and the protein kinase C pathway (PKC) (Zheng et al., 2017). These abnormalities can result in the formation of ROS thereby inducing lung damage (Yan, 2018). Recent studies reported that mitochondrial ROS and excessive amount of NADH in the lung that likely contributed to polyol pathway activation in a diabetic rat model (Wu et al., 2017). In addition, interaction of AGEs with their receptors (RAGEs) can affect various pathways and induce oxidative stress, inflammation, fibrosis, and cell death in different cells including endothelial cells, AT2 cells, bronchial epithelial cells, alveolar macrophages, and endothelial cells (Morbini et al., 2006; Byun et al., 2017). Binding of AGES to extracellular matrix (ECM) proteins can create anchoring sites for proteins such as albumin, collagen, and elastin that lead to ECM thickening and damage to endothelial cells. Indeed, the clinical severity of COVID-19 is associated with both endothelial damage and pulmonary microvascular thrombosis (Ciceri et al., 2020; Gattinoni et al., 2020; Pitocco et al., 2020; Varga et al., 2020). So, pre-existing complications in DM patients including increased ROS production in diabetic cells and endothelial cell damages could be a risk factor for adverse outcomes in patients with COVID-19 (Pitocco et al., 2020; Whyte et al., 2020). Viral infection similar to DM can induce ROS in infected cells and pre-existing ROS in diabetic cells can provoke virus-induced ROS production (Chernyak et al., 2020). It has been reported that imbalance in redox homeostasis in respiratory viruses-infected cells can trigger different pathological pathways in these cells and induce tissue damage (Khomich et al., 2018). In general, appropriate levels of ROS are required for immunological responses and viral clearance. However, excessive ROS levels have destructive effects, not only on virus-infected cells but also on healthy cells in the lung by oxidizing lipid membranes, cellular proteins, and nucleic acids (Wu et al., 2013; Wang J. Z. et al., 2020). Respiratory viruses can induce enzyme activities that have a role in ROS generation including xanthine oxidase and dinucleotide phosphate oxidases. In addition, antioxidant enzymes activity such as SOD, glutathione (GSH), and catalase (CAT) can be affected during respiratory viruses infection (Khomich et al., 2018). Therefore, ROS production in diabetic cells infected by viruses is significantly higher compared to non-diabetic infected cells (Oliveira et al., 2020). As described previously, in diabetic cells compared to non-diabetic cells, increased expression of ACE2, endothelial cells dysfunction, and high glucose level in diabetic cells can facilitate virus entry and replication, as well as low antioxidant level and high ROS level (Oliveira et al., 2020).

### Induced Inflammation in Infected Diabetic Lung Cells

In addition to oxidative stress, DM/obesity can induce inflammation in the lung. It has been demonstrated that both in chemically induced diabetes and in spontaneously type 1 diabetic mouse model, DM can promote inflammatory cell infiltration into the lung interstitium (Yang et al., 2011). Additionally, expression of tumor necrosis factor (TNF)-α, a robust pro-inflammatory cytokine, was significantly higher in diabetic lung compared to normal lung. Another study showed that TNF-α, IL-1β, and IL-6 levels are higher in the lung tissue of diabetic rats compared to that of healthy rats. Moreover, the NF-κB pathway was activated in the lung tissue of diabetic rat (Zhang et al., 2015). The NF-κB pathway can be activated by hyperglycemia and oxidative stress, and induce expression of pro-inflammatory genes (Evans et al., 2002; Baker et al., 2011). It has been reported that the level of inflammatory cytokines including IL-6, TNF-α, and IL-1β is higher in the lung of patients with DM and/or obesity (Talakatta et al., 2018). Pre-existing DM-induced inflammation can therefore exacerbate virus-induced inflammation in diabetic/infected cells compared to non-diabetic and healthy cells, thereby contributing to the cytokine storm observed in severe COVID-19 manifestations (Zhou et al., 2021).

It has been shown that respiratory viruses induced-ROS can also induce inflammation through activation of the NF-κB pathway or through induction of cell death and subsequently trigger macrophage activity and inflammation (Khomich et al., 2018). Pre-existing immune and endothelial cell dysfunction, high level of ROS, and elevated inflammatory cytokines in diabetic cells may set the stage for exacerbated inflammatory cytokines production following virus infection (Oliveira et al., 2020). Moreover, active viral replication would drive primary inflammatory responses, which lead to apoptosis or pyroptosis as a result of cytokine/chemokine production and cellular damage (Fu et al., 2020). Since the plasma level of IL-1β, an essential cytokine during pyroptosis is elevated in SARS-CoV-2 patients (Huang et al., 2020), pyroptosis has been suggested as another pathogenic mechanism involved in COVID-19 infected lung cells (Yang, 2020). When a pathogen like a virus invades the cell, pattern-recognition receptors (PRRs) such as NOD-like receptors protein 3 (NLRP3) on the cell membrane, recognize pathogen-associated molecular patterns (PAMPs) on the surface of the pathogen and bind to the precursor of caspase-1 (pro-caspase-1) through the adaptor protein ASC (Apoptosis-associated speck-like protein containing a caspase recruitment domain) in the cell. The newly formed multiprotein complex can activate caspase-1, which participates in innate immunity by activating pro-inflammatory cytokines like pro-IL-1β and pro-IL-18. Caspase-1 also plays an essential role in cell perforation by incising and polymerizing Gasdermin family members such as GSDMD. Consequently, the cellular content would be released into the extracellular space due to extensive cell perforation, resulting in inflammation (Yang, 2020). It has been reported that DM/obesity may activate the NLRP3 inflammasome cascade in diabetic cells and exacerbate COVID-19 symptoms. So, pre-existing NLRP3 inflammasome activation and increased release of inflammatory cytokines in diabetic cells may be one of the mechanisms that can provoke virus-related damages (Lambadiari et al., 2020). Secondary inflammatory responses occur when adaptive immunity appears and when the neutralizing antibodies are generated. Binding of the virus-anti-S-IgG complex to Fc receptors present on monocytes/macrophages membranes (Fu et al., 2020) would promote the production and secretion of pro-inflammatory cytokines and chemokines like IL-6, IFNγ, MCP-1, and IP-10 into the blood stream of patients (Huang et al., 2020; Zhang et al., 2020).

## Cumulative Effects of Destructive Mechanisms at the Cellular Level

**FIGURE 1:**
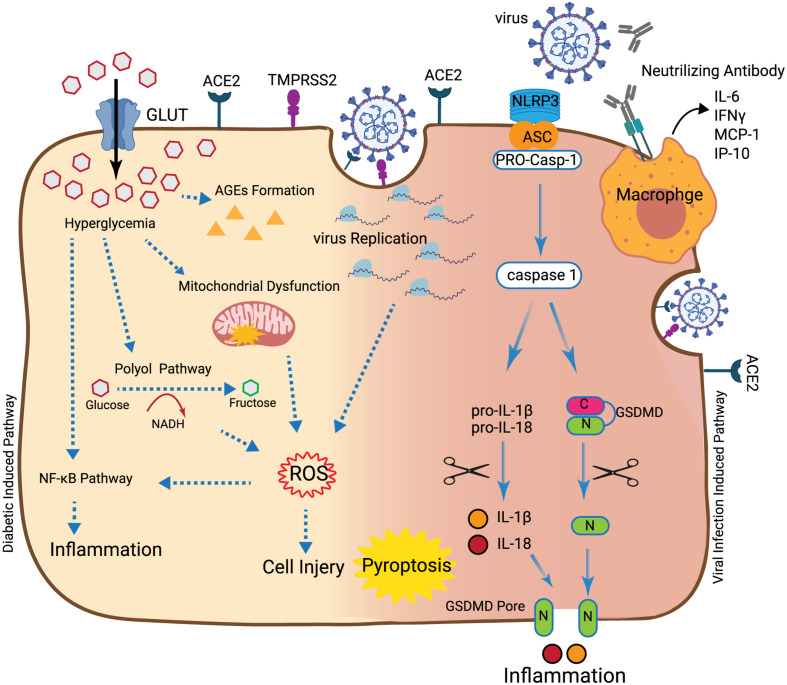
Synergic destructive effects of diabetes and SARS-CoV-2. In the hyperglycemia situation, accumulation of glucose into the cell can induce mitochondrial dysfunction, production of AGEs and polyol pathway activation that can induce ROS. Production of ROS can lead to cell injury and also inflammation through activation of the NFκB pathway. Also, after the virus enters into the cell through ACE2, the initial virus replication can lead to ROS induction. Once more, ROS can induce inflammation through activation of the NFκB pathway or induction of macrophage activity. Recognition of the virus by NLRP3 at the cell surface leads to binding of NLRP3 to the pro-caspase-1 through the ASC. This connection activates caspase-1 which leads to the cleavage of pro-inflammatory cytokines like pro-IL-1β, pro-IL-18, and GSMD. This results in the production of mature IL-1β and IL-18 and N-terminal products, respectively. N-terminal products located into cell membranes result in the creation of the GSMD pore. Following cell perforation and release of the cellular content into the extracellular space, inflammation, cell swelling, and pyroptosis can occur. After appearance of the adaptive immune response, generation of neutralizing antibodies and binding of macrophage virus receptors to the virus, cytokines, and chemokines such as IL-6, IFNγ, MCP-1, and IP-10 are produced and secreted into the bloodstream of patients.

As described earlier, COVID-19 symptoms can be exacerbated in patients with DM and obesity (Guan et al., 2020; Huang et al., 2020; Yang et al., 2020). DM through different mechanisms including mitochondrial dysfunction, polyol pathway, and AGEs formation can cause cell damage and induce oxidative stress and inflammation (Figure 1; Zheng et al., 2017). DM can affect SOD, CAT, and GSH enzymatic activities and facilitate ROS production (Forgiarini Junior et al., 2010). DM can induce inflammation through activation of the NF-κB pathway (Baker et al., 2011). It has been reported that the level of inflammatory cytokines including IL-6, TNF-α, and IL-1β is higher in the lung of patients with DM and/or obesity (Talakatta et al., 2018). Virus infection can induce oxidative stress and inflammation in the cells through different mechanisms. Virus replication in infected cells can induce ROS (Cecchini and Cecchini, 2020). Similar to what is observed for DM-induced oxidative stress, antioxidant enzyme activities including CAT, SOD, and GSH are decreased during the virus infection. Induced-ROS in infected cells can induce inflammation through activation of the NF-κB pathway (Fernandes et al., 2020). However, cumulative effects of two main pathogenic events including oxidative stress and inflammation in infected/diabetic cells may contribute to increased cell damage and tissue injury. So far, it is not clear whether DM-induced oxidative stress and inflammation have direct effects on the initiation and/or amplitude of virus-dependent pathogenic mechanisms. During virus infection in patients with DM/obesity, interactions between deregulated antioxidant enzymes activity and activated inflammatory pathways such as the NF-κB pathway may be the likely cause for the reported virus-dependent complications. Inhibition of oxidative stress by quercetin and inflammation by cortisone in diabetic patients play indeed a crucial role in attenuating COVID-19 complications (Azimi et al., 2020; Colunga Biancatelli et al., 2020).

Further studies need to be carried out to determine the role of pre-existing oxidative stress and inflammation present in the lung of diabetic patients in the context of SARS-CoV-2-infection. To that end, it is important to understand the interaction of AT2 cells, the target of SARS-CoV-2, and their tightly associated mesenchymal cells which play the role of a niche for AT2 stem cells; this title is developed and discussed through following section.

## Adult Lung Regeneration in Terms of Cell-Cell Interactions

### An Overview of AT2-Resident Stromal Cells Interaction

Lipofibroblasts (LIFs) are lipid-droplet-containing interstitial fibroblast which functions to transfer triglycerides to the AT2s. AT2 cells are also considered as stem/progenitor cells capable of self-renewal and differentiation toward alveolar epithelial type 1 (AT1) cells (Youk et al., 2020). They interact with resident lung mesenchymal cells which control the maintenance of these stem cell properties. Therefore, LIFs are proposed to constitute the mesenchymal niche for AT2 stem cells. However, LIFs are heterogeneous populations which have been isolated based on the expression of different markers. Platelet derived growth factor receptor alpha (Pdgfrα^Pos^), Axin2^Pos^ and Fibroblast growth factor 10 (Fgf10^Pos^) are three sub-lineages reported to be located close to AT2 cells. However, these three markers do not label clearly distinct populations of mesenchymal cells raising the possibility that these three populations may at least partially overlap. For example, 74% of resident stromal Axin2^Pos^ cells in the alveolar region are also expressing Pdgfrα. Axin2^Pos^ Pdgfrα^Pos^ mesenchymal cells are called mesenchymal alveolar niche cells (MANC) and are located close to AT2 cells (Zepp et al., 2017). The interaction between AT2 and stromal cells has been functionally tested using the alveolosphere model, which is a co-culture of AT2s with MANC in growth factor reduced Matrigel. In this assay, the MANCs have been reported to support AT2 self-renewal and differentiation toward the AT1 lineage (Barkauskas et al., 2013; Zepp et al., 2017).

### Fgf10^Pos^ Cells Maintain the Self-Renewal and Differentiation of AT2 Stem Cells

*Fgf10* is expressed early during lung development in the distal mesenchyme (Bellusci et al., 1997). Fgf10 acts in a paracrine fashion on the adjacent epithelium expressing Fgfr2b. Fgf10 promotes epithelial survival and branching morphogenesis. A subpopulation of *Fgf10*-expressing cells labeled at embryonic day 12.5 serve as progenitor for LIFs at later stages of lung development. Additionally, in postnatal lungs, around 30% of LIFs express *Fgf10* (Al Alam et al., 2015; Chu et al., 2021). Fgf10 itself, through an autocrine effect, appears to contribute to their differentiation (Bellusci et al., 1997). Using the alveolosphere assay, Fgf10^Pos^ cells maintain the self-renewal and differentiation of AT2 stem cells and appear to be distinct from the previously described MANCs (Taghizadeh et al., 2021).

### The Resident Mesenchymal Niche Is Impacted Following Massive AT2 Injury

**FIGURE 2:**
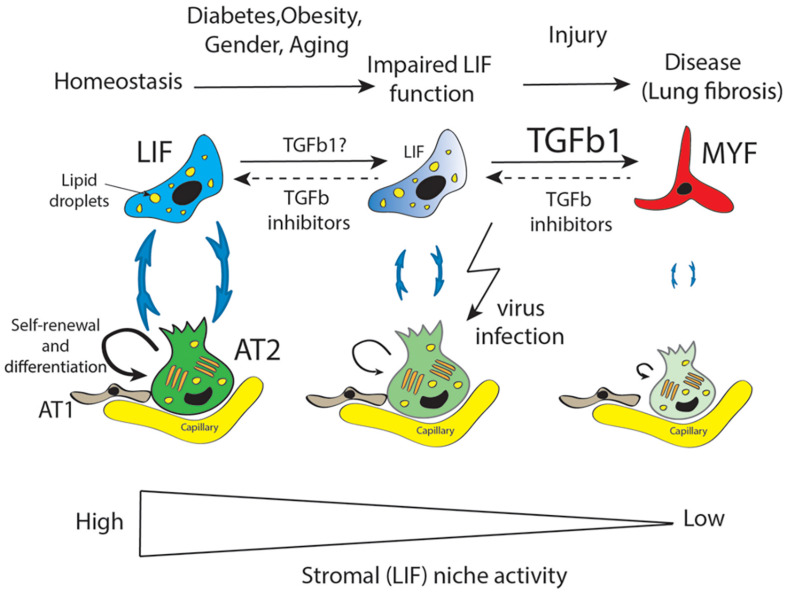
LIF is an important mesenchymal population supporting AT2 stem cells in terms of self-renewal and differentiation to AT1 cells. LIF cells provide triglycerides to AT2 for the elaboration of surfactant. Diabetes, obesity, aging, and gender are proposed to be the main factors impacting negatively the mesenchymal/stromal (LIF) niche activity resulting in decreased AT2-LIF interaction. Against this background, further injury to the lung leads the impaired LIF to transdifferentiate to activate myofibroblast (MYF). Accumulation of MYF is a main characteristic of lung fibrosis which over time, in particular in old males, leads to failure of lung function. Moreover, AT2 cells are the main target of virus infection which drastically reduces their function in terms of surfactant protein production. TGFβ is a causative growth factor for fibrosis induction and maintenance. We propose that TGFβ inhibitors could be instrumental in restoring impaired LIF function in the context of diabetes, obesity, gender, and aging as well as in the MYF to LIF transdifferentiation in the context of fibrosis.

Repetitive AT2 damage results in scarring and accumulation of activated MYF in the lung, which is the main characteristic of lung fibrosis (Figure 2). Activated myofibroblasts originate from multiple sources (Phillips et al., 2004; Kim et al., 2006; Rock et al., 2011; El Agha et al., 2014) and express high level of smooth muscle actin (SMA) as well as extracellular matrix proteins. Lung fibrosis is a chronic, progressive, and lethal disease considered as a long-term complication of COVID-19. It has been reported, using the bleomycin-induced fibrosis mouse model, that LIF transdifferentiate to activate MYF (El Agha et al., 2017). The contribution of the LIF-Fgf10^Pos^ cells to fibrosis formation is still unclear and will require further investigation using our recently developed *Fgf10^CreERT2^* knock in mouse line (Chu et al., 2021).

AT2 expressing ACE2 are thought to be the main target of COVID-19. However, there is also the possibility that the LIFs themselves may be a target for COVID-19 (Kruglikov and Scherer, 2020). Adipocytes (from the white adipose tissue) in COVID-19 patients express higher level of ACE2 compared non-infected patients. In addition, obesity enhances ACE2 expression in adipocytes. As LIFs share commonalities with adipocytes (for example, their high lipid droplet content, their dependence on Fgf10 signaling for their formation), it is possible that LIFs also represent cellular targets for COVID-19. In this situation, both the AT2 stem cells and their niche would be impacted, resulting in irreversible lung damage and lethality (Figure 2; Lv et al., 2021).

From a mechanistic point of view, it was reported that COVID-19 induces the expression of transforming growth factor β (Tgf-β), a growth factor causative for fibrosis formation and maintenance (Lal, 2010). On the therapeutic side, two drugs, Pirfenidone (TGFβ1 inhibitor) and nintedanib (pan-tyrosine kinase FGF receptor inhibitor) have been approved for patients suffering from idiopathic pulmonary fibrosis (IPF), a form of fibrosis which does not resolve and develops in old age, mostly in men. In addition, it has been demonstrated that DM and obesity were associated with IPF (Kruglikov and Scherer, 2020). Therefore, at least two of the three aggravating conditions described in the context of COVID-19 are also met in the context of IPFs raising the possibility that Pirfenidone and nintedanib could be instrumental to limit fibrosis progression post-COVID-19 infection.

Interestingly metformin, a first line antidiabetic drug, has been proposed to enhance MYF to LIF transition in the bleomycin-induced fibrosis murine model as well as *in vitro* using primary IPF fibroblasts (Kheirollahi et al., 2019). This drug may be useful for COVID-19 patients who are not suffering from diabetes.

## Linking Metabolic Dysfunction, Gender and Age With the Activity of the Resident Mesenchymal Niche

It was previously reported that aging is impacting the capacity of the stromal niche to support AT2 cell proliferation following pneumonectomy in mice (Paxson et al., 2011). Using the alveolosphere assay, it has been reported that obesity and gender also impact the capacity of resident mesenchymal cells defined as (Cd31Cd45Epcam)^Neg^Sca1^Pos^, isolated from *Ob/Ob* and Wild type male and female mice, to support AT2 stem cell self-renewal and differentiation (Taghizadeh et al., 2021). Resident stromal cells from *Ob/Ob* mice were less capable to support organoid formation compared to resident mesenchymal cells from wild type mice. It has been found that resident mesenchymal cells from male mice were less capable to support organoid formation compared to resident mesenchymal cells from female mice. The combined presence of obesity and male gender led to the complete loss of resident mesenchymal cells functionality. This is strikingly identical to what is observed in the human population, where males and obese/diabetic patients are the most impacted by COVID-19. To our knowledge, this observation represents the first clear link between metabolic diseases, gender and aging and a critical mesenchymal cell type needed for the proper maintenance of AT2 stem cells. How aging, gender and obesity can impact the resident mesenchymal cells is completely unknown and will be important to define *per se* using state of the art techniques. In the future, we propose that the use of the *in vitro* alveolosphere assay to test drugs capable of activating resident mesenchymal cells from *Ob/Ob* male mice will allow to designing new therapies to treat fibrosis complications in male, aged and diabetic patients who survived COVID-19-infection.

## Conclusion

Diabetes mellitus/obesity may exacerbate SARS-CoVID-2 infection through different mechanisms. Immune cell dysfunction, increased expression of ACE2 on lung cells, induction of virus replication, delay in virus clearance after infection, and high glucose concentration are just some of the mechanisms that can exacerbate SARS-CoVID-2-related pathogenic mechanisms in diabetic cells compared to non-diabetic cells (Oliveira et al., 2020). DM/obesity as a pre-existing risk factor can induce different pathogenic mechanisms including oxidative stress and inflammation at the cellular level and promote virus-related damages in infected cells. Oxidative stress and inflammation are the two key linking pathogenic cascades in DM/obesity and SARS-CoVID-2-induced mechanisms (Albulescu et al., 2020). So, potential therapies for the reduction of inflammation and oxidative stress in patients with DM may have a critical role in attenuating COVID-19-related infections and mortality. In addition, DM/obesity can negatively impact the ability of the lung mesenchymal niche to support AT2 stem cells. We propose that this failure is causative for fibrosis formation in surviving COVID-19 patients. Moreover, *in vitro* lung models such as alveolar organoids can be instrumental to investigate the impact of DM, obesity and gender on lung mesenchymal niche and provide a platform for testing new drugs capable of restoring their function.

## Author Contributions

MN-K, ST, AF, and AE wrote the manuscript. SV prepared the table and read the manuscript. RH, MT, AK, CC, and J-SZ read the manuscript and revised it. SB and YT designed the manuscript, read the manuscript, revised it, and approved the final version. All authors contributed to the article and approved the submitted version.

## Conflict of Interest

The authors declare that the research was conducted in the absence of any commercial or financial relationships that could be construed as a potential conflict of interest.

## Abbreviations

DM: Diabetes mellitus
SARS-CoV-2: severe acute respiratory syndrome coronavirus 2
COVID-19: coronavirus disease 2019
ARDS: acute respiratory distress syndrome
CAMs: cell adhesion molecules
VEGF: vascular endothelial growth factor
NK: natural killer
ACE2: angiotensin-converting enzyme 2
AT2: alveolar epithelial type 2
GSH: glutathione
CAT: catalase
PRRs: pattern-recognition receptors
NLRP3: NOD-like receptors protein 3
PAMPs: pathogen-associated molecular patterns
MDA: malondialdehyde
SOD: superoxide dismutase
AGEs: advanced glycation end products
PKC: protein kinase C
ROS: reactive oxygen species
TNF- α: tumor necrosis factor
ECM: extracellular matrix proteins
LIFs: Lipofibroblasts
AT1: alveolar epithelial type 1
IPF: idiopathic pulmonary fibrosis
MANC: mesenchymal alveolar niche cells
SMA: smooth muscle actin.
